# The double role of GABAergic system in systemic tumors: an updated review

**DOI:** 10.3389/fonc.2025.1570380

**Published:** 2025-08-18

**Authors:** Xueyang Yang, Qianhe Zu, Luxuan Wang, Chunhui Li, Lijian Zhang

**Affiliations:** ^1^ Department of Neurosurgery, Clinical Medicine College, Affiliated Hospital of Hebei University, Hebei University, Baoding, Hebei, China; ^2^ Clinical Medicine College, College of Basic Medicine, Hebei University, Baoding, Hebei, China; ^3^ Clinical Medicine College, Department of Neurological Function Examination, Affiliated Hospital of Hebei University, Hebei University, Baoding, Hebei, China; ^4^ Postdoctoral Research Station of Neurosurgery, Affiliated Hospital of Hebei University, Hebei University, Baoding, Hebei, China

**Keywords:** GABA, systemic tumors, autophagy, tumor microenvironment, therapeutic targets

## Abstract

The GABAergic system is the main inhibitory nervous system. In addition, GABA has been reported to affect tumor growth and its expression differs between tumor tissue and normal tissue. However, the impact of GABAergic system on tumor progression is context-dependent. The dual potential of the GABAergic system to exert either pro-tumor or anti-tumor effects is fundamentally shaped by the distinct histological features of the neoplasm. The complex components of the GABAergic system and signaling pathways involved might be responsible for this phenomenon. In this study, we reviewed the role of the GABAergic system in promoting or inhibiting tumorigenesis in different organ systems and summarized the possible signaling pathways regulated via GABAA receptor-associated protein (GABARAP). We also discussed the possible role of GABARAP in tumor progression through the regulation of autophagy. Additionally, this study suggest novel therapeutic approach targeting the GABAergic system in the treatment of tumors.

## 1 Introduction

GABA, as an inhibitory neurotransmitter, plays a crucial role as a metabolite in various cells within the human body. It has been shown that GABA is predominantly synthesized in neurons via a specialized metabolic pathway closely integrated with the tricarboxylic acid (TCA) cycle, requiring the coordinated activity of multiple enzymes including glutamate decarboxylase (GAD), pyridoxal phosphate-dependent enzymes, and mitochondrial transporters (1–3). Emerging studies highlight the pleiotropic roles of the GABAergic system, extending far beyond its canonical function in the central nervous system (CNS). Current research reveals its critical involvement in maintaining pancreatic β-cell homeostasis, modulating hormone secretion dynamics, and orchestrating immune responses in inflammatory diseases (4, 5). Previous evidences have demonstrated that GABAergic systems play important roles in tumor proliferation, metastasis, stemness, and tumor microenvironment (6). However, the oncogenic regulatory effects of GABAergic signaling have been found to be contradictory, suggesting bidirectional modulation of tumorigenic processes through cell type-specific mechanisms. Thus, a better understanding of the GABAergic signaling could provide novel insights into cancer treatment (**Tables 1**, **2**).

**Table 1:** The GABA system promotes tumor growth.

Type of tumors	GABA energy system	Signaling pathways	References
Glioma	GABA-A	Src-EZH2-OCT4	(10)
Medulloblastoma	ABAT	ABAT/H3K4ac.	(13)
Head and neck cancer	GABA	CCND2/BCL2L1	(14)
Oral squamous cell carcinomas	GAD	β-catenin and MMP7/ p38 MAPK	(15, 16)
Nasopharyngeal carcinoma	GAD	miR-24-3p/CYTOR	(17)
Thyroid and parotid tumor	GABA	cyclic AMP/ ERK 1/2	(19, 20)
Lung cancer	GABA-A	JAK1/STAT6/TAMs	(21)
Lung adenocarcinoma	GABRA3	JNK/AP-1/ MMP-2 /MMP-9	(22)
Breast cancer	GABA and GABA-A	Ca2/PKC/CREB	(23)
	GABAB1e	PTPN12/EGFR/AKT	(25)
Basal-like breast cancer	GABA-π	ERK1/2	(24)
	ABAT	Ca2+-NFAT1	(27)
Gastric cancer	GABA-A	ERK1/2	(33)
Hepatocellular carcinoma	GABA-A-θ/α3/γ2	GABRA3	(34–36)
Pancreatic ductal adenocarcinoma	GABA-π	Ca2+	(37)
	GABRP	MEK/ERK	(38)
		KCNN4-- Ca2+/NF-κB	(39)
Colorectal cancer	GABABBR1	–	(42)
	GABA	cMYC	(43)
Prostate cancer	GABABR	EGFR-ERK1/2-- MMPs	(45)
	GABBR1	GRP	(46)
Castration-resistant prostate cancer	GABA	PI3K-PKC Є	(131)
	GABAα1	EGFR-Src	(91)
Renal cell carcinoma	GABA-B	MAPKs/ERK1/2	(49)
Ovarian cancer	GABA-π	MAPK/ERK	(50)

**Table 2:** The GABA system inhibits tumor growth.

Type of tumors	GABA energy system	Signaling pathways	References
Glioma	GABA-A/ GABA-Aα1	mir-139-5p	(51, 52)
	GAD1/GAD2/ABAT	–	(57, 58)
NSCLC	GABARAP	NIX/ ubiquitin-binding protein p62	(59)
Breast cancer	GABARAP	AKT/mTOR	(62)
	GABA-Aα3	AKT	(17)
Gastric cancer	GABA-Aβ3	Bcl-2/BAX/Caspase axis	(63)
Hepatocellular carcinoma	GABA-A-β2/γ2	alpha-fetoprotein	(65, 66)
	GABA-A/ρ1	miR-183-5p	(6, 68)
	GABA-B	cAMP/p62	(67)
	ABAT	miR-183-5p	(69)
Cholangiocarcinoma	GABA	MMP-2 /MMP-9	(71)
	GABA/GABA-B	JAK/STAT3	(72)
	GABA-B	GSK3α/β-STATA	(73)
	GABA	cAMP-ERK1/2	(74)
Pancreatic cancer	Gabra3	AKT/mTOR	(78)
Colorectal cancer	GABABR	cAMP- ERK/CREB- cIAP2	(79)
	GABA-B	Hippo/YAP1-EMT	(80)
		GSK-3β/NF-κB	(81)
Chondrosarcoma	GABA-B	MAPK/PI3K/AKT/mTOR	(82)
Renal cell carcinoma	ABAT	HNF4A	(83)

Autophagy is an essential cellular event for the maintenance of cell homeostasis. This process is tightly regulated and involved in virous physiological processes, including metabolism, membrane trafficking, and immune and inflammatory processes (7). The dysregulation of autophagy is typically triggered by multilevel factors including amino acid starvation, decreased insulin levels, reduced ATP and hypoxia. These dysregulations might lead to a range of pathological conditions such as oncogenesis (8). The GABA A-receptor-associated protein (GABARAP), a family of proteins comprising GABARAP, GABARAPL1, GABARAPL2, and GABARAPL3, have been identified as key regulators of autophagy (9). Moreover, GABARAPs showed multifaceted oncogenic regulatory functions across diverse malignancies. Current researches highlight their potential as clinical biomarkers and potential therapeutic targets (10, 11). However, the precise mechanism of GABARAPs in tumors and other diseases is still unclear and requires further investigation.

**Figure 1:**
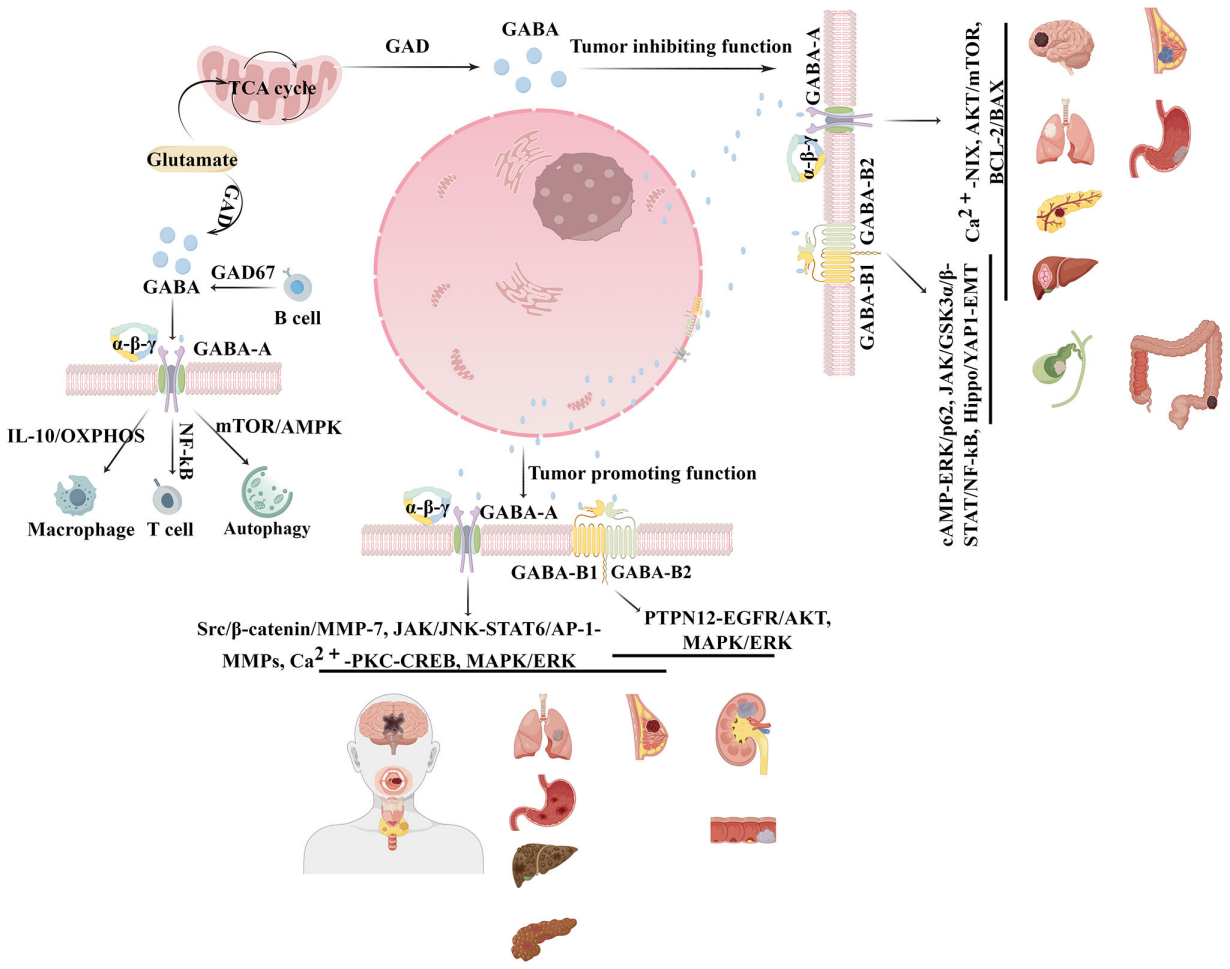
Schematic of GABAergic signaling in oncobiology via regulating immune cells in the tumor microenvironment and autophagy.

This review discussed the context-dependent duality of GABAergic signaling in oncobiology, delineating its paradoxical tumor-promoting and suppressive outcomes across heterogeneous malignancies. It also summarized recent advances in research focusing on the GABAergic system and autophagy, which highlight the GABAergic system as a potential target for the treatment tumors (**Figure 1**).

## 2 The GABAergic system promotes tumor growth

### 2.1 Intracranial tumor and head and neck cancer

Head and neck squamous cell carcinomas (HNSCCs) represent the sixth most prevalent malignancy globally. Evidences have indicated that gamma-aminobutyric acid (GABA) plays pivotal roles in neurotransmission, regulation of cell differentiation, proliferation, and tumorigenesis in HNSCCs. However, the precise mechanisms of GABAergic system in HNSCCs are still unknown. Gliomas are one of the most common types of neurological malignancies derived from glial cells. The 5th edition of the World Health Organization (WHO) classification of glioma was according to specific molecular alterations (12). Results obtained from a large-scale analysis of multiple omics datasets showed substantial differences in gene expression, methylation and miRNAs associated with neurotransmission in adult gliomas, suggesting that gliomas might warrant reclassification based on neurotransmitters (13). Additionally, a unique neurotransmitter-related microenvironment has been identified (14). The results obtained by Anja Smits and her colleagues showed a distinct variation in the subunit proteins of the GABA-A receptor in glioma, which affects the clinical outcome of glioma (15). Evidences have indicated that propofol, a GABA-A receptor agonist, increased the Src expression and further promote the palmitoylation of EZH2 and OCT4 which facilitates the development of glioma (16). However, propofol has been shown to inhibit glioma cell growth and invasion in other studies (17). One possible explanation for this discrepancy might be the GABA-A receptor and its subunits. In addition, there is considerable variability in the expression of GABA-A receptors and their subunits in different types of gliomas and glioma cell lines. Further research is needed to elucidate the possible interactions between these subunits of the receptor (18, 19). Medulloblastoma (MB) is a malignant brain tumor that mainly affects children. There is evidence showed the abnormal activation of GABA in MB. In addition, GABA transaminase (ABAT) has been observed to maintain MB cell viability and facilitate leptomeningeal metastasis formation (20).

Previous studies have reported that GABA might play an important role in regulation the proliferation and apoptosis of HNSCCs via promoting the expression of cyclin D2 (CCND2) and B-cell lymphoma 2-like 1 (BCL2L1) (21). Glutamate decarboxylase 1 (GAD1) expressed in GABAergic neurons is a rate-limiting enzyme in the production of GABA. Studies have demonstrated that GAD1 is highly expressed in oral squamous cell carcinomas (OSCC) and is intricately linked to the metastasis and invasion of oral cancers via β-catenin and MMP7 (22). In addition, it has been observed that GABA and its receptor agonists can promote the proliferation of OSCC cells through the activation of p38 MAPK (23). GAD1 was found to be upregulated in non-metastatic nasopharyngeal carcinoma (NPC) biopsies (24). There is also evidence showed that Cytoskeleton Regulator RNA (CYTOR) could regulate GAD1 via targeting miR-24-3p. And this gene has been shown to promote the proliferation, migration and invasion of NPC cell lines (25). In addition, it has been reported to enhance tumor invasion and could serve as an important prognostic indicator of recurrence and metastasis in early and partially advanced nasopharyngeal carcinoma patients (26). Moreover, studies have shown that the expression level of GABA receptors are elevated in tumor tissue compared to normal thyroid and parotid tissue. These findings suggest that the GABAergic system may be involved in the etiology of thyroid and parotid gland tumors (27, 28).

### 2.2 Thoracic neoplasms

GAD1 expression is higher in lung cancer than in normal tissue. It is negatively correlated with overall survival in lung adenocarcinoma patients. And overexpression of GAD1 might result in an increase in GABA levels (29). One previous study also showed that tumor-derived GABA could bind to the GABA-A receptor on the surface of macrophages, resulting in activation the JAK1/STAT6 pathway in tumor-associated macrophages (TAMs), which promotes macrophage polarization towards the M2 phenotype and further facilitate the tumor progression (30). Their findings suggested that tumor-derived GABA might have complex mechanisms in promotion the tumorigenesis not only directly promote the tumor cells proliferation but also modulate the tumor-microenvironment. In addition, GABRA3, a type A receptor subunit of GABA, has been reported to induce the expression of MMP-2 and MMP-9 through activation of the JNK/AP-1 pathway, further promoting lymphatic metastasis of lung adenocarcinoma (LUAD) (21).

Integrated metabolomic and transcriptomic profiling in breast cancer has identified ALDH1A3 as a definitive cancer stem cell (CSC) marker, strongly associated with tumor initiation, therapy resistance, and metastatic potential. Notably, dysregulated GABAergic metabolic reprogramming was found to increase the expression of ALDH1A3 and promote breast cancer metastasis (31). Alanine aminotransferase 2 (GPT2), a key enzyme bridging amino acid and GABA metabolism, exerts pro-metastatic effects through increasing Ca2+ flux through GABA and GABA-A receptors and activation of the PKC-CREB signaling pathway (32). However, the proliferation and invasion of breast cancer cell lines remains to be investigated. Evidence has showed that the pi subunit of the GABRP is expressed in the basal-like breast cancer (BLBC) subtype and may be associated with the development of breast cancer metastasis. This receptor has previously been reported to be involved in the ERK1/2 signaling pathway, which may contribute to cancer cell migration (3). In addition to the GABA-A receptor, studies have demonstrated that phosphorylation at sites Y230 and Y404, subunits of the GABA-B receptor GABAB1e, interact with PTPN12, which affects the EGFR/AKT signaling pathway and promotes the malignancy of breast cancer cells. GABAB1e is also expressed in other tumor cell lines as demonstrated by studies (33, 34). It is possible that, in the absence of relevant *in vitro* data, there may be a relationship between gamma-aminobutyrate aminotransferase (ABAT) and GABA levels. It has been suggested that GABA levels may be associated with the activity of GABA receptors, which could potentially contribute to the progression of BLBC (35). Clinical and epidemiological data have been interpreted to suggest that GABA could have significant prognostic value in breast cancer, with a novel risk score model indicating the potential for predicting survival outcomes for breast cancer patients (36, 37).

Despite advances in the treatment of these diseases, lung cancer and breast cancer brain metastases remain major challenges in field of oncology. The pathogenesis of these diseases remains poorly understood, with limited knowledge of the underlying molecular pathways and cellular mechanisms involved. Existing literature suggests that lung and breast cancers establish suitable conditions for metastasis prior to the onset of metastatic disease (38, 39). Furthermore, some studies have shown that brain metastases tissues exhibit a GABAergic energy phenotype similar to that observed in neurons and have suggested that enhancing GABA metabolism may promote proliferation of tumor tissues (40). These observations provide a rationale for further investigation of the potential relationship between the GABAergic energy system and brain metastasis.

### 2.3 Abdominal neoplasms

Comparative tissue analysis revealed higher expression level of GABA and GAD in tumor tissue than adjacent tissues, suggesting that the GABAergic system might be a potential therapeutic target for gastric cancer (41). Conversely, studies have shown that GABA could activate the ERK1/2 signaling pathway via the GABA-A receptor, which in turn promotes the proliferation of gastric cancer cell lines (42). However, this finding has not been validated by *in vivo* experiments. Moreover, evidences also suggest that GABA could promote the cell proliferation in hepatocellular carcinoma (HCC) via the GABA-A-θ subunit. However, purified GABA fails to influence the proliferation of tumor cell lines after receptor knockout (43). Further studies have shown that GABA can promote the proliferation of malignant liver cancer cells through GABA-Aα3 in a dose-dependent manner (44). In addition to the direct stimulation of GABA receptors in liver cancer, Nova-1, a pro-cancer RNA binding protein (RBP), can directly bind to GABAγ2 to promote tumor growth *in vivo* (45). Pancreatic ductal carcinoma (PDAC) has the highest mortality rate among the most common malignancies. A study based on genome-wide cDNA microarray analysis of PDAC revealed that the GABA receptor P-subunit (GABRP) was overexpressed in PDAC cells. Their results also showed that the MAPK/ERK cascade could promote the proliferation and metastasis of PDAC cells through the influence of Ca2^+^ (46). In addition, GABRP has also been shown to activate the MEK/ERK signaling pathway, thereby promoting metastasis (38). Furthermore, GABRP interacts with KCNN4 and stimulates the Ca_2_^+^-NF-κB signaling pathway, leading to increase tumor cell growth, macrophage aggregation and tumor invasiveness (39). Those studies also provided new insights into the immunomodulatory process involved in the initiation and development of PDAC.

EphB6 is a tyrosine kinase receptor that has been implicated in colorectal cancer (CRC). Previous studies have shown a correlation between EphB6 expression and the development of CRC in patients (47). Recent *in vivo* experiments have indicated that EphB6 deficiency may be a contributing factor to the development of colorectal cancer (CRC) tumors. This potential mechanism involves the upregulation of synaptosomal-associated protein 25 (SNAP-25), which has been observed to increase GABA levels within the tumor microenvironment (48). GABAB receptor expression is highly prevalent in CRC cell lines, and animal studies have shown that GABA can increase the stability of the cMYC protein, inhibit its ubiquitination degradation, and promote the proliferation and migration of colorectal cancer cells (49). In addition, GABABBR1 could facilitate CRC proliferation and invasion through targeted regulation by miRNAs such as miR-106a/b, miR-20a/b, and miR-17 (50). Moreover, previous studies have also shown that the GABA-A receptor is expressed in prostate cancer and is involved in the proliferation of prostate cancer cells (51). GABABR has been shown to activate the EGFR-ERK1/2 signaling pathway. This leads to increased expression of MMPs, which contribute to prostate cancer invasion and migration (52). It has also been observed that GABA could induce gastrin-releasing peptide (GRP) secretion via GABBR1, which in turn promotes prostate cancer invasion and migration (53). Nevertheless, the present body of research fails to deliver a thoroughgoing elucidation of the interrelationship between the proliferative and migratory capacities of cells and the potential cross-talk among the signaling pathways influenced by diverse receptors and small molecules. Studies have shown that the enzyme GAD65, a key enzyme in the synthesis of the inhibitory neurotransmitter GABA, might promote the progression of castration-resistant prostate cancer (CRPC) by increasing GABA diversion, and this process may be mediated by the PI3K-PKC ϵ signaling axis (54). Moreover, there is evidence that GABAα1 could activate the EGFR-Src signaling pathway in a paracrine or autocrine manner, thereby promoting the progression of CRPC. This effect of GABA on tumor growth through secretion may be a new direction for studying the mechanism of GABA on tumors (55). Interestingly, GABA regulates the biological behavior of renal cell carcinoma (RCC) and ovarian cancer (OC) through the same signaling pathway via different receptors (56, 57).

In recent years, a growing body of research has suggested that electrical activity may play an important role in the initiation and development of tumors (58). Studies have shown that GABAergic signaling could promote the development of melanoma through its effect on the basic electrical activity between cells (59). Furthermore, this study has shown that upregulation of GAD1 is closely associated with the ability of primary tumors to form, suggesting that GAD1 may serve as a valuable prognostic marker (60).

## 3 The GABAergic system inhibits tumor growth

### 3.1 Intracranial tumor

The mechanism of the GABAergic system in glioma development remains controversial. A number of studies have indicated that endogenous GABA could potentially suppress glioma cell proliferation via GABAA receptor subunits, while others have suggested a contrary effect, proposing that GABA may in fact promote glioma progression (61). However, the detailed mechanism of action of GABA *in vivo* remains to be elucidated. Recent studies have shown that GABA-A receptor centered miRNA can directly or indirectly regulate glioma growth. In addition, miRNAs such miR-139-5p affected glioma malignant biological behavior via targeting gamma-aminobutyric acid A receptor alpha 1(GABRA1) (62). Furthermore, propofol has been shown to play an anti-tumor role by regulating non-coding RNAs. For example, miRNA-206 and lncRNA have been observed to inhibit glioma cell proliferation by blocking or decreasing the expression of the PI3K/AKT signaling pathway and c-Myc/GSTM3 (17, 63). A previous study showed that GABA-A receptors are exclusively expressed in low-grade and anaplastic gliomas. The observed loss of expression in glioblastoma might be regulated by miRNAs (64). In recent years, a significant body of research has demonstrated the existence of a potential bidirectional interaction between the gut microbiota and the brain (65). Some scientists have hypothesized the existence of a regulatory mechanism by which the gut microbial community influences neurotransmitter levels, with subsequent effects on glioma development (66). The GABAergic stroma has been shown to inhibit glioma growth (67). A study of the GBM database showed that GAD1, GAD2 and ABAT levels were decreased in mesenchymal GBM and that this decrease was associated with poor prognosis. This suggests that a reduction in GABA production or an increase in metabolism may be associated with GBM progression (68). However, further research is needed to confirm this mechanism of GABA signaling in glioma.

### 3.2 Thoracic neoplasms

Pharmacological activation of GABA-A receptors has been shown to induce autophagy by activating the GABA-A receptor-associated protein (GABARAP), the mitochondrial receptor NIX and utilizing the ubiquitin-binding protein p62, which inhibits the proliferation of non-small cell lung cancer (NSCLC) cells and brain metastases while reducing the toxicity of radiotherapy (69). In addition, the intravenous anesthetic propofol has been shown to inhibit lung cancer invasion and metastasis via the GABA-A receptor pathway (70). There is also evidence that the GABA receptor may have multiple roles in the biological behavior of tumor cells as well as in the signaling pathway in lung cancer (6). The findings obtained from Zhang and colleagues suggested that the pattern of GABA receptor gene phenotype expression may be involved in the regulation of tumorigenesis. And the expression of GABA receptor may be not only promising genetic therapeutic targets but may also serve as valuable prognostic markers for NSCLC (71). However, further research is needed to elucidate the detailed mechanism by which the GABAergic system exerts its effect on lung cancer. In contrast to NSCLC, GABARAP is also able to regulate epithelial-stromal transformation (EMT) in BC via the AKT/mTOR signaling pathway, thereby inhibiting the proliferation and invasion of breast cancer cells (72). Interestingly, Kiranmai Gumireddy et al. showed that GABA-Aα3 could activate the AKT signaling pathway in breast cancer and promote tumor cell migration and invasion, while mRNAs and key enzymes that produce GABA-Aα3 were only expressed in some breast cancer cell lines. And GABA-Aα3 in the form of mRNA editing has been shown to inhibit the AKT signaling pathway and breast cancer metastasis (73). This study may provide a new avenue of research to investigate the various mechanisms of action of the GABAergic system in tumor tissues.

### 3.3 Abdominal neoplasms

Gastric carcinoma (GC) is a malignant neoplasm that imposes a substantial global burden. GABA and its receptors have been showed to play vital roles in the occurrence and progression of GC (74). Recent studies demonstrated that Epberberine (EPI) could cause cell cycle arrest and induce cell apoptosis via Bcl-2/BAX/caspase axis by targeting GABA-Aβ3 receptor (75). However, this study did not extend its investigation to the potential consequences of GABA-Aβ3 stimulation by GABA or EPI on GC cell lines. A recent study has suggested that molecular subtypes associated with GABA receptor activation may be able to predict the prognosis of patients with gastric cancer (76).

Minuk et al. proposed that alterations in the expression of GABAergic signaling in the liver might contribute to the pathogenesis of hepatocellular carcinoma (HCC) and inhibit the proliferation of malignant liver cell lines via GABA-A receptors β2 and γ2 (77, 78). In addition, studies have shown that GABA-B receptor agonists could inhibit the migration of HCC cell lines via cAMP and p21 (79). Notably, the results obtained from Chen et al. showed that GABA could suppress the migration, invasion and metastasis of HCC cells through GABA-A receptors (80). Their findings emphasized that GABA receptor might represent as potential therapeutic target for liver cancer treatment. Bioinformatic analysis showed that the expression of ABAT was lower in HCC tissues than in normal or adjacent non-cancerous tissues. And the overexpression of ABAT could inhibit the proliferation, migration and invasion of HCC cells (81). In addition, ABAT has been proposed as a prognostic indicator in HCC. An animal experiment based on the TCGA database also verified the change in ABAT expression in HCC (82). In contrast, some researchers have suggested that the GABA-A receptor subunit ρ1 is associated with a short overall survival (OS) in HCC and may also be a potential prognostic indicator in HCC (83). However, the complex mechanism of action of the GABAergic system in HCC represents a significant obstacle to the realization of its treatment for HCC.

Previous research showed that GABA inhibited the invasion and migration of cholangiocarcinoma (CCA) cell lines by suppressing the activity and expression of MMP-2 and MMP-9 (84). And their further studies have revealed that GABA binds to GABA-B receptors, which may inhibit the proliferation of cholangiocarcinoma cells via JAK/STAT3 (85). In addition, recent research has demonstrated that GABA-B receptor agonists have the ability to inhibit the proliferation of bile duct cancer cells via the GSK3α/β-STATA pathway and its downstream targets in cholangiocarcinoma from diabetic patients (86). Furthermore, findings by Giammarco Fava et al. indicated that GABA can suppress the proliferation of cholangiocarcinoma cell lines via upregulation of the cAMP-ERK1/2 signaling pathway (87). CCA is an aggressive tumor, with the majority of patients presenting with advanced disease at the time of symptom onset. The current available systemic therapies have limited efficacy and short survival (88). Therefore, it is imperative to identify new therapeutic targets. Existing studies have shown that the GABA-B receptor could significantly inhibit tumor cell growth in CCA. Therefore, the GABA-B receptor may be a promising therapeutic target for cholangiocarcinoma. However, no further studies have been conducted to demonstrate whether the GABA-B receptor exerts the same inhibitory effect *in vivo*.

Contrary to previous findings, Banerjee et al. demonstrated that GABA monotherapy is as effective as gemcitabine in pancreatic cancer, affecting multiple signaling pathways and effectively reversing nicotine-induced drug resistance (89). It is noteworthy that baclofen, a GABA-B receptor agonist, has also been observed to promote pancreatic cancer progression and potentially increase drug resistance (90). This phenomenon may be due to the difference in affinity between GABA and the GABA-A and GABA-B receptors in pancreatic cancer. However, previous studies have shown that the GABA-A receptor could also facilitate the progression of pancreatic cancer. The multiple roles of the GABA system in pancreatic cancer are also reflected in the current state of research into the effects of the GABA system on tumor tissue (91). Further research is still needed. The results of this study are similar to previous studies which showed that the combination of the COX-2 inhibitor celecoxib and GABA can significantly inhibit pancreatic cancer growth (92). However, the study did not clearly elucidate whether GABA can independently play a role in inhibiting tumor growth and the effect of inhibition. In addition, miR-92b-3p can inhibit the expression of AKT/mTOR and JNK signaling pathways by inhibiting Gabra3 (GABA-A receptor subunit) and ultimately inhibit the proliferation, migration and invasion of PC cells (93).

Colorectal cancer (CRC) is the another most commonly diagnosed abdominal neoplasms. The GABAergic system also plays a significant role in colorectal cancer (CRC) by influencing tumor growth and immune responses. Recent studies have shown that Lactobacillus plantarum can inhibit cAMP-dependent ERK/CREB phosphorylation through the GABA receptor (GABABR), promote the expression of apoptosis protein 2 (cIAP2) and ultimately induce apoptosis of tumor cells (94). Further studies have shown that GABAB receptors may inhibit CRC progression by regulating the Hippo/YAP1 pathway and EMT (95). In addition, GABA-B has been shown to inhibit the proliferation of colorectal cancer cell lines by regulating the GSK-3β/NF-κB signaling pathway (96). The above studies on CRC suggest that GABA-B is more highly expressed in CRC tissues and has a greater inhibitory effect on CRC. However, comparative studies of the GABA-A receptor in CRC are still limited and further verification of its specific mechanism of action is required.

### 3.4 Other neoplasms

Chondrosarcoma is a rare type of bone cancer that develops in cartilage cells. It is the most common bone cancer in adults that usually begins in the bones, but can sometimes occur in the soft tissue near bones. Chondrosarcoma happens most often in the pelvis, hip and shoulder. More rarely, it can happen in the bones of the spine or extracranial skull base (97). Chondrosarcoma arises from neural crest cells and the GABAergic system plays a key role in the development of the nervous system. Therefore, the GABAergic system may play an important role in the development and progression of chondrosarcoma. There is evidence that changes in intracellular calcium can facilitate programmed death in chondrosarcoma cell lines by inhibiting the MAPK and PI3K/AKT/mTOR signaling pathways through GABA-B receptor-related calcium channels (98). The prognoses of chondrosarcomas are strongly correlated with histologic grading. Generally, chondrosarcoma has a high incidence of local recurrence and metastasis despite surgical resection, which is associated with poor prognosis. However, the studies focused on the elucidation of the relationship between the GABAergic system and high-grade chondrosarcomas are limited. The spatial heterogeneity of GABA-related signaling pathways in anatomically distinct chondrosarcomas remains unclear, meriting in-depth exploration. In contrast to the direct stimulation of GABA-B observed in the renal cell carcinoma (RCC) cell line, there is evidence that ABAT is significantly decreased in patients with RCC and can significantly inhibit cancer cell proliferation and migration following overexpression (99). This suggests that the GABAergic system may have a more profound mechanism in RCC, and no studies have yet demonstrated the role of GABA receptors between RCC cells.

As discussed above, the opposite outcomes of GABAergic elements in different types of cancers could be attributed to several factors. The downstream molecular pathways of GABAergic signaling in different tumors are diverse such as PI3K-PKC signaling axis, EGFR-ERK1/2 signaling pathway, c-Myc/GSTM3, AKT signaling pathway, Bcl-2/BAX/caspase axis etc. And the variation in the subunit proteins of the GABA-A receptor might be another contributing factor. Variability in the expression of GABA-A receptors and their subunits in different types of tumor cells has been reported previously. Moreover, the expression level of GABA in tumor tissue and adjacent tissues was diverse. And the GABA might have complex mechanisms in modulating tumorigenesis not only by directly regulate the tumor cells proliferation but also regulate the tumor-microenvironment indirectly.

## 4 GABARAPs and autophagy

Autophagy is a major intracellular degradation process that transports cytoplasmic components to lysosomes for degradation. It is a central molecular pathway for the maintenance of cellular and organismal homeostasis (100). Since its discovery in the 20th century, autophagy has been extensively studied and its association with various diseases has gradually emerged (101). The present study explores the process of GABA-A receptor cluster formation and maintenance, induced by presynaptic terminals during the formation of synaptic connections. This study suggests that autophagy-associated proteins may play a role in the process of transporting GABA-A from the cell surface, following endocytosis, to the autophagy-derived compartment for degradation. This finding suggests the presence of a hitherto unrecognized link between GABA receptors and autophagy (102). It has been established that GABARAPs are distributed across the entirety of the human body. The study of the central nervous system (CNS) has been identified as providing a reference point for further studies of GABARAPs in other systems (103). For example, there is evidence that GABA can attenuate intestinal epithelial apoptosis caused by enterotoxin-producing Escherichia coli (ETEC) through the AMPK autophagy pathway (104). In osteoarthritis (OA), GABARAP has demonstrated efficacy in preserving the viability of bone mesenchymal stem cells (BMSCs), whilst concomitantly promoting their osteogenic potential. This effect is achieved through the initiation of autophagy-related pathways (105). In addition, autophagy has been studied in the context of metabolic, pulmonary, renal, infectious, musculoskeletal and ocular diseases (100). It should also be noted that certain pathways, which have been observed to influence autophagy via associated GABA receptor-related proteins, also play a substantial role in the development and genesis of tumors.

As research progresses, an increasing body of evidence suggests that autophagy-related genes may play a pivotal role in the development of tumors (106). Autophagy is generally considered to have a dual function in the process of tumor initiation and development, with the potential to either inhibit or promote tumorigenesis. Recent studies have shown that the conflicting roles of autophagy in tumors may be influenced by various factors, including the specific oncogenes and tumor factors involved. This may be related to different oncogenes and tumor factors (107). However, the specific mechanism of autophagy in different tumors is still not well understood. Further investigation of the complex molecular regulatory mechanisms and the different roles of autophagy, GABA and its receptor-related proteins may be necessary. GABARAPs is a critical component of the mammalian autophagy-related protein Atg8 (108). GABAA receptor-associated protein-like 1 (GABARAPL1) is a member of the GABARAPs family. Studies have shown that knocking down GABARAPL1 in breast cancer cell lines can lead to inhibition of autophagy, which is regulated by the mTOR and AMPK signaling pathways. This in turn affects the growth of tumor cells (109). In addition, studies by Douglas S. Grunwald and Paula Szalai et al. have shown that GABARAPs are essential for the proper regulation of autophagy initiation and progression, as well as partial autophagy in relation to LC3s (110, 111).

In recent years, GABARAPL1 has been identified as a novel autophagosome marker. A study in triple negative breast cancer (TNBC) has shown that high expression of GABARAPL1 is significantly associated with poor prognosis in TNBC. Inhibition of GABARAPL1 has been shown to induce apoptosis in TNBC cells and inhibit metastasis (112). Furthermore, research has demonstrated that the GABARAPL1 gene is subject to regulatory influence from CREB-1, otherwise referred to as CREB binding protein 1. This regulatory association has been observed in specific breast cancer cell lines (113). In addition to TNBC, GABARAPL1 overexpression in nasopharyngeal carcinoma (NPC) can induce autophagosome formation, reduce HIF-2α, and then promote apoptosis of nasopharyngeal carcinoma cells to inhibit tumor cell growth (114). And overexpression of GABARAPL1 has been shown to inhibit tumor cell proliferation in hepatocellular carcinoma (HCC) cell lines and may be associated with prognosis (115). There is evidence that a long non-coding RNA (lncRNA), nuclear-enriched abundant transcript 1 (NEAT1) variant 1 (NEAT1v1), could promote autophagy through GABARAP and lead to radio-resistance of hepatocellular carcinoma (HCC) cells (116). In prostate cancer, GABARAPL1 is regulated by the androgen receptor (AR), which affects the proliferation of prostate cancer cells (117, 118). As previously mentioned in the treatment of NSCLC, GABARAP-NIX could induce autophagy, thereby controlling tumor progression and reducing radiation toxicity (68). Glioma is one of the most common malignant tumors of the nervous system. And autophagy-related genes (ARGs) play an important role in glioma occurrence, progression, and treatment. Previous have identified GABARAP as one of the ARGs which is associated with the development of glioma (119). The preceding research posits the hypothesis that the effects of GABA on particular tumor tissues may be the consequence of its impact on autophagy.

## 5 GABA regulates immune cells in the tumor microenvironment

The tumor microenvironment (TME) is a complex and dynamic milieu that plays a crucial role in cancer progression and response to treatment. It is composed of various components, including tumor cells, immune cells, stromal cells, and extracellular matrices, which interact and mutually regulate each other (120). The notion that GABA is exclusively of glial origin, or derived from tumor tissue, is demonstrably fallacious. Recent findings have revealed that B cells have the capacity to induce the synthesis and secretion of GABA following activation by foreign stimulation, thereby constraining anti-tumor immunity (121). In addition, there is evidence showing that the GABA-A receptor is associated with the development and function of the immune system. CD8+ T cells are end effectors of cancer immunity. Most forms of effective cancer immunotherapy involve CD8+ T cell effector function (122). GABA has been shown to regulate the proliferation and migration of T-cells and to influence the growth and metastasis of tumor cells. This process might be initiated by GABA stimulation of the GABA-A receptor on the surface of CD8+ T cells (123). CD4+ T lymphocytes have been implicated in antigen presentation, cytokine release, and cytotoxicity, suggesting their contribution to the dynamics of the TME (124). It is noteworthy that GABA-A receptor activation could reduce the number of Treg cells, thereby inhibiting the invasion and migration of lung cancer. However, studies have also shown that GABA can inhibit CD4+ T cells and promote the proliferation of Treg cells, limiting anti-tumor immunity (70, 125). The different phenotypes of macrophages have different effects on tumors. Research suggests that GABA can enhance IL-10 and oxidative phosphorylation (OXPHOS), the expression of related proteins, to accelerate the transformation of the anti-inflammatory macrophage phenotype and promote its infiltration, thereby facilitating tumor progression. At the same time, it can inhibit CD8+ T cells, thereby impeding the anti-tumor immune response (121). In addition, GABA-A receptors have been shown to affect peripheral blood mononuclear cells and antigen-presenting cells, including dendritic cells (DCs) (125, 126). Although existing studies have demonstrated that GABA can directly affect immune cells through GABA-A receptors, inhibit anti-tumor immunity and promote tumor cell growth and metastasis, the mechanism of its immune escape in the tumor immune microenvironment remains unclear. Some studies have suggested that GABA signaling may influence the immune microenvironment in tumors, including pancreatic cancer (91). In addition, GABRP is positively correlated with the density of macrophages in the tumor. Macrophages are thought to promote tumor progression by inhibiting immune cells in the tumor immune microenvironment (127). Although studies have shown that GABA can influence the efficacy of immunotherapy, and research on the GABAergic system in the immune system has increased in recent years, the specific mechanism by which GABAergic signaling affects the tumor immune microenvironment remains unclear. One of the most important questions is how the GABAergic system is induced in tumors and what are the specific roles of downstream targets of the GABAergic system and immune cells during tumor progression and metastasis. In conjunction with the above studies, certain non-coding RNAs have been shown to influence the expression of GABA-A receptor subunits. GABA-A plays a central role in immune cells, suggesting that this pathway may warrant further investigation. In addition, related ion channels may play a crucial role in the GABAergic system. For example, Ca2+ ion channels have been shown to play a central role in breast cancer progression, pancreatic cancer drug resistance and immune cell development (125).

## 6 Novel therapeutic targeting GABAergic system

A growing body of research indicates that the GABAergic system plays a pivotal role in various tumor tissues through different mechanisms of action. Thus, a novel therapeutic approach to tumors involving GABAergic system expression regulation has been developed and validated in select tumor types. It is hypothesized that drugs targeting the GABAergic system could prove effective in treating tumors. A plethora of GABA receptor-related drugs have been developed, including benzodiazepine analogs, that can activate GABA-A receptors, thus modulating melanoma progression and enhancing the efficacy of radiation and PD-1 therapy. This provides a reference for the use of psychotropic drugs as anti-tumor drugs (128). Meanwhile, it has been demonstrated that GABA-related receptors are able to influence the radio resistance of tumor cells via autophagy-related genes. These results suggest that GABA-related drugs may not only have a direct effect on tumor treatment, but may also be used as an adjuvant drug to increase the therapeutic efficacy of specific tumors. However, a critical evaluation of preceding studies indicates that the impact of GABA receptor stimulation and suppression on cancerous cell proliferation may be paradoxical. Consequently, the utilization of this approach in tumor treatment remains constrained by limitations such as the conflicting effects of propofol in glioma. Furthermore, as one of the most commonly used anesthetics, the potential tumor-promoting or tumor-inhibiting effects of propofol during surgery must be considered (17). The heterogeneity and intricacy of GABA receptors, in conjunction with the variations amongst distinct tumor tissues, could potentially elucidate the equivocal outcomes pertaining to the utilization of GABA antagonists and agonists within anti-tumor pharmaceuticals. In comparison to receptor agonists and inhibitors, the potential applications of ABATs and GADs drugs that affect GABA metabolism appear to be more promising. Studies have shown that the FDA-approved drugs bilobalide and vigabatrin, which can regulate GADs and ABAT, have inhibitory effects on gastric and breast cancer BMS cells (107, 108). The development of advanced gene editing technologies, most notably the clustered regularly interspaced short palindromic repeats- CRISPR associated protein (CRISPR-Cas) system, has enabled large-scale and systematic investigations of genetic interactions (129). Diehl et al. identified interactions among paralogous genes including GABARAP- MAP1LC3B and GABARAP-GABARAPL2 which suggested the utility of CRISPR-based methods to investigate the therapeutic targets involving GABARAP (130–133). Moreover, the autophagy flux assays results showed the essential function of the Atg8 family and identify GABARAP sub-family members (GBRPL1, GBRPL2) as primary contributors to PINK1/Parkin mitophagy and starvation autophagy (14). These novel technological approaches provide great assistance for us to explore their potential molecular mechanisms and related molecular pathways.

## 7 Conclusions

A number of studies have showed the dual potential of the GABAergic system on tumor progression. There is an urgent need for further investigation into the safety of GABAergic system-targeted drugs. The regulatory factors centering around GABA, which play a pivotal role in various physiological and potentially pathological processes, warrant in - depth investigation. This paper reviews the possible mechanisms of the GABA signaling system to promote or inhibit tumor initiation and development, as well as their impact in immunity and autophagy. However, the precise mechanism by which the GABAergic system contributes to tumor metastasis remains to be elucidated. Furthermore, the confinement of existing research to specific cell lines implies that distinct cell lines derived from the same tumor may generate divergent research outcomes.
